# Systematic Comparison of CRISPR and shRNA Screens to Identify Essential Genes Using a Graph-Based Unsupervised Learning Model

**DOI:** 10.3390/cells13191653

**Published:** 2024-10-04

**Authors:** Yulian Ding, Connor Denomy, Andrew Freywald, Yi Pan, Franco J. Vizeacoumar, Frederick S. Vizeacoumar, Fang-Xiang Wu

**Affiliations:** 1 Central for High-Performance Computing, Shenzhen Institute of Advanced Technology, Chinese Academy of Sciences, Shenzhen 518055, China; yl.ding2@siat.ac.cn (Y.D.); yi.pan@siat.ac.cn (Y.P.); 2 Division of Biomedical Engineering, University of Saskatchewan, Saskatoon, SK S7N 5A9, Canada; 3 Cancer Research Department, Saskatchewan Cancer Agency, Saskatoon, SK S7N 5E5, Canada; franco.vizeacoumar@usask.ca; 4 Division of Oncology, University of Saskatchewan, Saskatoon, SK S7N 5E5, Canada; connor.denomy@usask.ca; 5 Department of Pathology, University of Saskatchewan, Saskatoon, SK S7N 5E5, Canada; andrew.freywald@usask.ca; 6 Department of Computer Science and Control Engineering, Shenzhen University of Advanced Technology, Shenzhen 518055, China; 7 Department of Mechanical Engineering, Department of Computer Science, University of Saskatchewan, Saskatoon, SK S7N 5A9, Canada

**Keywords:** CRISPR, shRNA, gene expression, essential gene, graph-based model, consensus maximization

## Abstract

Generally, essential genes identified using shRNA and CRISPR are not always the same, raising questions about the choice between these two screening platforms. To address this, we systematically compared the performance of CRISPR and shRNA to identify essential genes across different gene expression levels in 254 cell lines. As both platforms have a notable false positive rate, to correct this confounding factor, we first developed a graph-based unsupervised machine learning model to predict common essential genes. Furthermore, to maintain the unique characteristics of individual cell lines, we intersect essential genes derived from the biological experiment with the predicted common essential genes. Finally, we employed statistical methods to compare the ability of these two screening platforms to identify essential genes that exhibit differential expression across various cell lines. Our analysis yielded several noteworthy findings: (1) shRNA outperforms CRISPR in the identification of lowly expressed essential genes; (2) both screening methodologies demonstrate strong performance in identifying highly expressed essential genes but with limited overlap, so we suggest using a combination of these two platforms for highly expressed essential genes; (3) notably, we did not observe a single gene that becomes universally essential across all cancer cell lines.

## 1. Introduction

Essential genes play critical roles in the survival and proliferation of all human cells [1,2]. Within human cancer cell lines, these essential genes have been identified for their ability to unravel disease mechanisms and catalyze the development of targeted therapeutics [3,4]. High-throughput functional genetic screening technologies have ushered in a new era, facilitating a comprehensive assessments of gene essentiality in the context of cancer cell survival [5,6]. In particular, the emergence of short hairpin RNA (shRNA)- and Clustered Regularly Interspaced Short Palindromic Repeat (CRISPR)-based genome-wide screening platforms has revolutionized the quest for therapeutic drug targets and diagnostic markers [7,8]. In contrast, the set of essential genes also offers an invaluable benchmark for assessing the comparative performance of diverse genetic screening methodologies [9]. Previous studies have shown that both screening platforms can be used to effectively separate essential and nonessential genes. shRNA refers to the technique of using the post-translational silencing of gene expression that occurs in response to the introduction of double-stranded RNA into a cell. It is considered to be an invaluable research tool for the rapid characterization of genes by suppressing the function of any chosen target gene with high specificity. Similar to shRNA, the CRISPR-Cas9 system is also widely used in genetic screening and biomedical research. Cas9 can induce DNA double-strand breaks at specific genomic loci with the help of a synthetic single-guild RNA, and targeting coding regions of genes can create frameshift insertion/deletion mutations that result in a loss-of-function allele. However, the results from these two screens show a low correlation, and the identified essential genes have a limited overlap [10]. This difference may be caused by several technical factors. First, the off-target effects of these two methods are different. It is well known that off-target effects and variable on-target efficacy are the main limitations of shRNAs [11]. CRISPR also causes off-target effects [12], but these disadvantages may be mitigated to some extent [13]. In addition, the phenotypes among the nontargeting areas identified in these two screens exhibited different distributions [14]. Second, the heterogeneity of the reagents used is the main reason for the reduction in the performance of shRNA-based screens and may also affect the efficiency of CRISPR-based screens [5]. Third, the difference between the timing of knockdown and knockout also affects the final results [10]. Another possibility is that the nonspecific effects of CRISPR and shRNA may result from differences in influence due to shRNA expression or CRISPR-Cas9 nuclease activity [15,16]. Above all, the intrinsic difference between gene knockdown and gene deletion may also contribute to this discrepancy [17]. While the difference between the two approaches is clear, it is unclear how the choice between shRNA or CRISPR may affect the results.

Several studies have compared the applications of CRISPR and shRNA genetic screening platforms in identifying essential genes and have arrived at different conclusions. Evers et al. [18] reported that CRISPR outperforms short hairpin RNA (shRNA) in the identification of essential genes based on the identification of 46 essential genes and 47 nonessential genes in the RT-112 cell line. They claimed that CRISPR had lower noise, more consistent activity across reagents, and fewer off-target effects. However, in the same year, Morgens et al. [10] reported a different conclusion by comparing 217 essential genes and 947 nonessential genes in the K562 cell line. The results showed that the precision of the two approaches [19] was similar, but the combination of those two screens could improve the performance. In addition, both screens revealed different biological processes associated with different Gene Ontology (GO) terms enriched in essential complexes [10]. Additionally, Hart et al. highlighted that shRNAs perform poorly when disrupting genes with low expression according to screening results in five human cell lines [9]; however, Morgens et al. further demonstrated that this phenomenon was caused by the low efficacy of the shRNA library, and this signature disappeared when they used a more effective hairpin design method [10,19]. These comparative studies have not found consistent results, and they all face some limitations. For example, comparisons are all based on the screening of a few cell lines, and it has been documented that gene essentiality changes in different cell lines [20], impairing the comparison of results. Additionally, the available gold standard essential genes are limited, which may also cause bias in the comparison results. Moreover, gene expression levels are often ignored, but it is important to consider essentiality scores based on gene expression levels. Several studies indicate the consistent conclusion that most essential genes had a higher probability to be highly expressed, and essentiality should be highly associated with gene expression level [9,21,22]. Finally, the comparisons are based on the screening results, which showed high false positive rates caused by off-target effects. These concerns necessitate careful and synthetic comparisons between shRNAs and CRISPR in the context of differentially expressed genes to identify essential genes in as many cell lines as possible.

In this study, we analyzed the essentiality scores from shRNA and CRISPR screens in 254 cell lines with their corresponding gene expression values and determined how the choice of screening platform affects the identification of essential genes with different gene expression levels. Because both platforms have a high false positive rate [23], we first developed a graph-based unsupervised machine learning model to correct this confounding factor. This model can identify common essential genes (and nonessential genes) from the 254 cell lines and obtain a more confident and robust gene-independent response to targeting. Unsupervised graph-based machine learning models can understand the structure of the data when it is given without any prior knowledge or guidance on what the data represent. In addition, it can uncover patterns or anomalies in data that were previously unknown. This is particularly useful in exploratory data analysis where you might not have a clear hypothesis about what you are looking for. Then, considering the specificity of each cell line and maintaining the true positive rate, the final essential genes were selected. This was performed by intersecting the experimentally derived gene essentiality score for each gene and the common essential genes identified with our graph-based unsupervised machine learning model. Finally, we used statistical methods to compare the performance of the two screening approaches in identifying essential genes under the context of different gene expression patterns in 254 cell lines. Our analyses suggested which screening platform should be chosen for the identification of essential genes across different gene expression levels.

## 2. Materials and Methods

### 2.1. Data Description

The data used in this study include the essentiality scores of each gene from CRISPR and shRNA in 254 matching cell lines, which were derived from DepMap (PMID: 28753430) [24]. The CRISPR essentiality score ranged from −3.897 to 2.274, with a standard deviation of 0.026 and a mean of −0.509, and the shRNA essentiality score ranged from −5.932 to 2.775 with a standard deviation of 0.057 and a mean of −0.164. A large positive essentiality score indicates a high confidence increase in cell growth after gene knockdown by shRNA or knockout by CRISPR (i.e., nonessential), whereas a small negative essentiality score indicates a high confidence decrease in growth rate (i.e., essential). Gene expression data were downloaded from the Cancer Cell Line Encyclopedia (CCLE) [25]. There were 19,177 genes in each cell line, and their expression ranged from 0 to 17.778. The mean expression value was 2.685, and the standard deviation was 0.083. The overlap among all the databases included 15,029 genes in 254 matching cell lines.

### 2.2. Overall Description of Our Workflow

**Figure 1:**
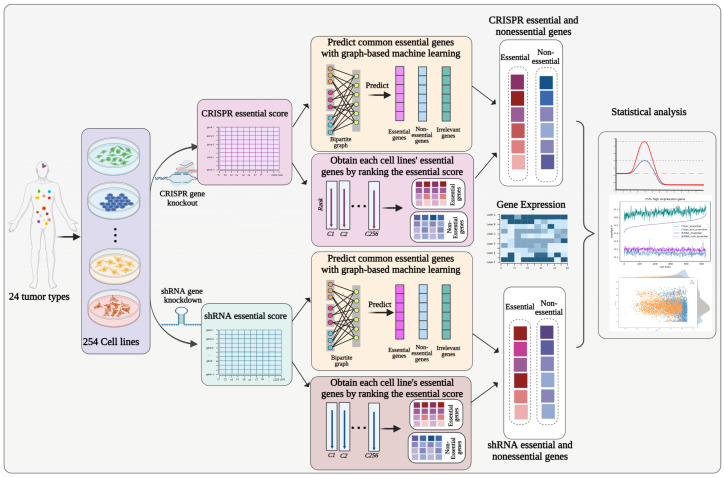
Workflow chart. Both the CRISPR essentiality score and shRNA essentiality score were obtained for each gene in each cell line. Then, a graph−based unsupervised learning model was applied to predict the common essential genes to correct the false positive samples, and the rank of the original essentiality score was applied to maintain the true positive rate. The intersection of essential genes (nonessential genes) identified via these two methods was considered to indicate the final essential genes (nonessential genes). The gene expression data were combined for further analysis via these two screening platforms.

Our goal was to compare shRNA and CRISPR for the identification of essential genes with respect to different gene expression levels in each cell line. The problem of identifying the essential genes can be described as follows: given *m* genes G = $\left\{g_{1},g_{2},\ldots ,g_{m}\right\}$ and *n* cell lines $\left\{c_{1},c_{2},\ldots ,c_{n}\right\}$, gene knockdown by shRNA and gene knockout by CRISPR were conducted on each cell line for each gene to obtain its essentiality score. For each gene $g_{i}\left(i=1,\ldots ,m\right)$ in each cell line $c_{j}\left(j=1,\ldots ,n\right)$, there are three possible states: (1) $g_{i}$ is deemed to be essential in cell line $c_{j}$; (2) $g_{i}$ is deemed to be nonessential in cell line $c_{j}$; and (3) $g_{i}$ is deemed to be an irrelevant (neither essential nor nonessential) gene in cell line $c_{j}$. As a result, for each cell line, all *m* genes can be classified into three classes: essential (E), nonessential (NE), and irrelevant (I). Thus, the correct estimation of which class a specific gene $g_{i}$ should belong to in a specific cell line, $c_{j}$, should be performed according to their essentiality score. After obtaining each gene’s essentiality via two different screening platforms, we combined the gene expression data with essential gene information and further applied statistical methods to compare and analyze those two platforms. The workflow is shown in Figure 1.

### 2.3. Graph-Based Unsupervised Learning Model

As described in the previous section, genes in each cell line can be classified into three groups. The *n* cell lines were subsequently divided into 3×n groups in total. Given a cell line $c_{j}$ for each gene $g_{i}$, it should belong to one of three groups: the essential group in the cell line $c_{j}$ (represented as $t_{\left(3j-2\right)})$, the nonessential group in the cell line $c_{j}$ (represented as $t_{\left(3j-1\right)})$, or the irrelevant group in the cell line $c_{j}$ (represented as $t_{\left(3j\right)})$. This approach formulates a group gene bipartite graph representation. In this bipartite graph, the group nodes are at the bottom of the graph, and the gene nodes are at the top of the graph. Each gene connects with one of the three groups in each cell line. Using gene $g_{1}$ as an example, it belongs to class $\left[t_{1},\ldots ,t_{\left(3j-2\right)},\ldots ,t_{\left(3n\right)}\right]$ because it is assumed to be essential in cell lines $c_{1}$ and $c_{n}$ but irrelevant in cell line $c_{j}$.

The m×v adjacency matrix *A* of this bipartite graph is defined as $a_{ik}=1$ if gene $g_{i}$ is linked with group $t_{k}$; otherwise, $a_{ik}=0$, where v=3×n is the total number of groups and *m* is the total number of genes. In each cell line, genes with the bottom 10% lowest essentiality scores were considered essential genes, whereas the genes with the top 10% highest essentiality scores were considered nonessential genes; the rest were considered irrelevant genes. Based on the essentiality score from the raw essentiality dataset, we initialize this matrix *A* and then estimate the conditional probabilities of gene $g_{i}$ being essential (nonessential or irrelevant). Let m×c matrix *U* collect these conditional probabilities. In addition, the conditional probabilities of each group $t_{k}\left(k=1,\ldots ,v\right)$ are also estimated and collected in the v×c matrix *Q*. Specifically, $U=(u_{iz})$ and $Q=(q_{kz})$ are defined as shown in Equation (1).

(1) $$\begin{array}{c} \begin{array}{c} u_{iz}=prob\left(g_{i}\;belongs\;to\;class\;z|g_{i}\right),\\ and\;q_{kz}=prob\left(t_{k}\;belongs\;to\;class\;z|t_{k}\right), \end{array} \end{array}$$

where c=3 and class *z* indicates one of the three groups: essential, nonessential, or irrelevant. Generally, group $t_{k}$ belongs to class *z* if the majority of genes in this group are from class *z*, whereas gene $g_{i}$ corresponds to class *z* if most of its linked groups belong to class *z*. Furthermore, let v×c matrix *Y* denote the initial class label matrix, where $y_{kz}=1$ if the group $t_{k}$ belongs to class *z* and otherwise $y_{kz}=0$. For example, if group $t_{1}$ belongs to the essential gene group, then the first row of *Y* is [1,0,0]; if $t_{1}$ belongs to the nonessential gene group, then the first row of *Y* is [0,1,0]. Then, the problem of estimating the conditional probability matrix *U* becomes the problem of optimizing the following objective function J(U,Q) with the constraints as shown in Equation (2).

(2) $$\begin{array}{c} \begin{array}{cc} & minJ\left(U,Q\right)=min[\sum_{z=1}^{c}\sum_{i=1}^{m}\sum_{k=1}^{v}a_{ik}{\left(u_{iz}-q_{kz}\right)}^{2}\\ & \;+\alpha \sum_{z=1}^{c}\sum_{k=1}^{v}{\left(q_{kz}-y_{kz}\right)}^{2}],\\ & s.t.\sum_{z=1}^{c}u_{iz}=1,\sum_{z=1}^{c}q_{kz}=1,u_{iz}\in \left[0,1\right],q_{iz}\in \left[0,1\right], \end{array} \end{array}$$

where $a_{ik}$ is the (i,k) element of matrix *A* of the bipartite graph. As each gene $g_{i}$ belongs to one of three groups in *n* cell lines, $\sum_{k=1}^{v}a_{ik}=n$. The objective function in Equation (2) includes two parts: the first part penalizes the deviation between essential group nodes and gene nodes. When $a_{ik}$ = 1, the difference between $u_{iz}$ and $q_{kz}$ should be small; otherwise, the first part is 0. To avoid overfitting in the first part, the second part is added to ensure that the final predicted results for the groups do not deviate too much from their original classes. α is the regularization coefficient to balance these two parts. The best *U* and *Q* minimize the objective function in Equation (2), which indicates that gene nodes and group nodes achieve maximal consensus.

### 2.4. Iterative Updating to Obtain Common Essential Genes

When we fix matrix *U*, the objective function in Equation (2) is quadratic in terms of the elements of matrix *Q*. The Hessian matrix with respect to *Q* is a diagonal matrix with diagonal elements $\sum_{i=1}^{m}a_{ik}+a>0$, which means that it is convex. At iteration *t*, the global minimum of the objective function is given in terms of *Q*. Then, we can obtain the updating equation for $q_{kz}$ as shown in Equation (3).

(3) $$\begin{array}{c} q_{kz}^{t}=\frac{\sum_{i=1}^{m}a_{ik}u_{iz}^{\left(t-1\right)}+ay_{kz}}{\alpha +\sum_{i=1}^{m}a_{ik}}. \end{array}$$

Similarly, when we fix matrix *Q* at iteration *t*, the objective function is quadratic in terms of the elements of matrix *U*. The corresponding Hessian matrix with respect to *U* is also a diagonal matrix with diagonal matrix elements $\sum_{i=1}^{m}a_{ik}>0$, which means that we can obtain the global minimum of J(U,Q). The updating formula for $u_{iz}$ is as shown in Equation (4).

(4) $$\begin{array}{c} u_{iz}^{t}=\frac{\sum_{k=1}^{v}a_{ik}q_{kz}^{\left(t-1\right)}}{\sum_{k=1}^{v}a_{ik}}=\frac{1}{n}\sum_{k=1}^{v}a_{ik}q_{kz}^{\left(t-1\right)}. \end{array}$$

We iteratively update *Q* and *U* according to Equations (3) and (4), respectively, until $\left|\right|U^{t}-U^{t-1}\left|\right|<\epsilon$. ϵ is a specified small positive number. In this way, group node *Q* can integrate the information from its neighboring genes during updating but without deviation from its original value *Y*, and *U* can receive information from different cell lines and propagate the information back to its neighboring genes.

### 2.5. The Final Essential Genes for Each Cell Line Were Obtained

To maintain the true positive rate, we took the intersection between the genes from the top *x*% of the experimentally derived essentiality scores in each cell line and the genes from the top *x*% of the predicted common essential genes with a high estimated probability as the final essential genes to conduct the next analysis. In this study, the percentage value *x* ranges from 10 to 40.

### 2.6. Statistical Analysis of shRNA- and CRISPR-Based Essential Genes with Different Expression Levels

We combined the expression of each gene in different cell lines and the essentiality of the gene obtained from the last section to statistically compare shRNA and CRISPR for the identification of essential genes with different gene expression levels. First, we analyzed the correlation coefficients between the shRNA essentiality score and gene expression, the CRISPR essentiality score and gene expression, and the shRNA essentiality score and CRISPR essentiality score with a Pearson correlation coefficient based on all the genes. We also compared shRNA and CRISPR via the correlation coefficient between the essentiality score and gene expression only for essential genes or nonessential genes. Then, for all the essential genes and nonessential genes in the 254 cell lines, we drew a scatterplot of the gene essentiality score and gene expression with marginal density plots. Based on the marginal density plot of gene expression, a threshold was set to identify essential genes. To visualize the distribution of essential (and nonessential) genes, the kernel density estimate was applied to visualize the joint distribution. After that, we focused on genes whose expression was low or high and compared the abilities of shRNAs and CRISPR to identify genes whose expression was low and those whose expression was high in each cell line. Finally, by comparing the essential and nonessential genes, we divided all the essential and nonessential genes in the 254 cell lines into 8 groups to determine their overlap and compare the expression of the genes in each group.

## 3. Results

### 3.1. Graph-Based Machine Learning Models Converge on Both shRNAs and CRIPSRs with the Same Trends

**Figure 2:**
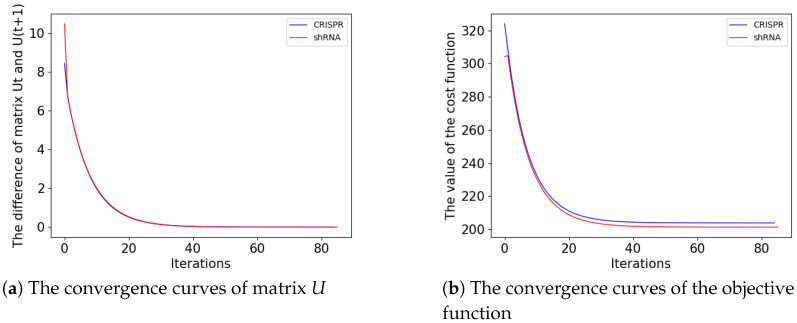
Convergence of the essential gene prediction model on shRNA and CRISPR. (**a**), The convergence curves of the difference between the matrix $U_{t}$ and $U_{\left(t-1\right)}$; and (**b**), The convergence of the objective function. In both of these figures, the red curve represents the shRNA common essential gene prediction model, while the blue curve represents the CRISPR common essential gene prediction model.

To understand the discrepancy between shRNA and CRISPR in calling out essential genes, we first attempted to generate a list of common essential genes. Current approaches for calling an essential gene largely depend on the frequency of its essentiality in multiple cell lines. To generate a robust list of essential genes, we applied a graph-based machine learning approach that can correct false-positive samples [26]. The parameter settings of the machine learning model are as follows. The first important parameter is α in Equation (2), which can indicate our confidence in the initial labels of the essential group nodes. According to the role of α in the updating rules of $q_{kz}$ in Equation (3), we set α to 1000. The second parameter is ϵ, which determines the endpoint for updating parameter $u_{iz}$, and it is typically a specified small positive number. We set this value to 0.0001 in this study. The third parameter is *x*%, which determines how many genes are considered essential (or nonessential) genes when we take the intersection to maintain the true positive rate and reduce the false positive rate. Generally, as the percentage *x*% decreases, fewer essential and non-essential genes are acquired, yet the ones that are obtained are of high quality and have a minimal rate of false positives, and vice versa. We choose *x*% in 10%, 20%, 30%, 40%, and to maintain high accuracy, *x*% is set as 10% in most of the following analyses. Based on these parameters, the convergence of our unsupervised graph-based machine learning model is shown in Figure 2. From these analyses, we found that both prediction models become stable after 80 iterations and that the convergence trends of matrix *U* in both models remain similar. For the objective function, shRNA converges at 201.321, whereas CRISPR converges at 203.832. Thus, the convergence trends of their objective functions are almost the same, indicating the validity of our prediction model.

### 3.2. Not a Single Gene Is Essential in All Cancer Cell Lines

**Figure 3:**
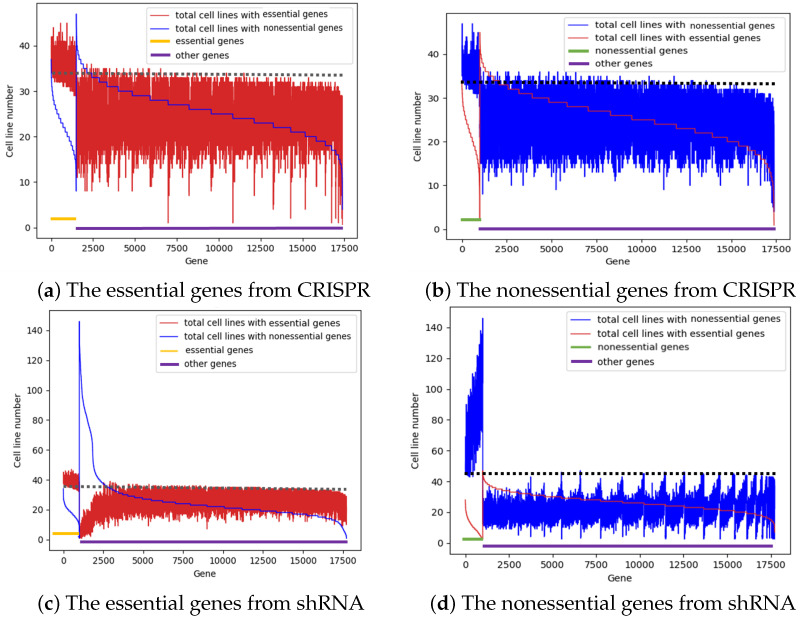
The essentiality of the top 1000 predicted essential genes and the top 1000 nonessential genes were evaluated via CRISPR and shRNA in 254 cell lines. The *x*-axis shows all the gene IDs, and the first 1000 genes are the predicted genes. The *y*-axis describes the total number of cell lines that are essential or nonessential for a specific gene. (**a**), The common essential genes identified via CRISPR. (**b**), The common nonessential genes identified via CRISPR. (**c**), The common essential genes identified via shRNA. (**d**), The common nonessential genes identified via shRNA.

To further evaluate the unsupervised prediction results, we next compared the top 1000 predicted essential genes and the top 1000 predicted nonessential genes from the graph-based machine learning models with the remaining genes. The predicted top 1000 essential genes for CRISPR and shRNA are detailed in Supplementary Table S1, while the predicted top 1000 nonessential genes for both screening platforms are included in Supplementary Table S2. For each gene, we evaluated its essentiality in the 254 cell lines by calculating the total number of cell lines in which they are essential and the total number of cell lines in which they are nonessential. For each cell line, we denote the bottom 10% of genes with a relatively low essentiality score as essential genes and the top 10% of genes with a relatively high essentiality score as nonessential genes. The essentiality of all the predicted essential genes or nonessential genes is shown in Figure 3. As shown in Figure 3a, the red curve shows that the 1000 common essential genes predicted via CRISPR have more cell lines showing essentiality than the rest of the genes. In fact, the maximum number of cell lines in which these genes are essential is only 40 out of 254 cell lines. However, these predicted results are different from those of simple voting methods, as a small number of predicted essential genes (highlighted below the dotted lines) had somewhat lower cell line numbers than the other genes. In Figure 3a, unlike those shown by the red curve, the blue curves, which represent the total number of cell lines with nonessential genes, have similar distributions of the predicted essential genes and the remaining genes. Figure 3b shows the predicted 1000 nonessential genes identified via CRISPR, and it is apparent that the predicted nonessential genes have more cell lines that are nonessential than the rest of the genes. Similar results were obtained with the analyses of the predicted genes according to the shRNA data (Figure 3c,d). In general, these data demonstrated that our prediction model is robust and that not a single gene is essential in all cancer cell lines.

### 3.3. Gene Expression and Gene Essentiality Are Positively Correlated According to Both shRNA and CRISPR Screening Methodologies

**Figure 4:**
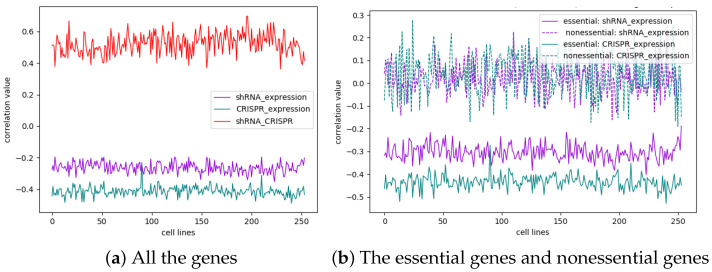
(**a**), The correlation coefficient between the essentiality score and gene expression in the original data for all the genes in each cell line. The purple curve corresponds to shRNAs with an average value of −0.263, and the green curve corresponds to CRISPR with an average value of −0.413. The red curve shows the correlation between shRNA and CRISPR with an average value of 0.523. (**b**), The correlation coefficient between the essentiality score and gene expression for essential genes and nonessential genes in each cell line. The average correlation coefficient between the shRNA essentiality score and gene expression was −0.304, the average correlation coefficient for CRISPR was −0.435, and the average correlation coefficient for nonessential genes on both shRNA and CRISPR was approximately 0.

We next investigated whether the gene essentiality score was correlated with gene expression. To investigate this phenomenon, we analyzed the Pearson correlation coefficient between the essentiality scores from shRNA/CRISPR and the gene expression data from CCLE for all the genes (Figure 4a). Interestingly, the correlation coefficient between the essentiality score and gene expression for shRNA was an average of −0.263, while the correlation coefficient of CRISPR was an average of −0.413. These findings indicate that both shRNA and CRISPR-mediated effects are weakly negatively correlated between the gene essentiality score and gene expression (a positive correlation between the gene essentiality score and gene expression because a negative essential score represents essentiality), with the correlation being slightly greater for CRISPR than for shRNA. We also explored the correlation between the essentiality scores of shRNA and CRISPR, which showed an average value of 0.523 (Figure 4a). This indicates that the essentiality scores from those two screening platforms have a medium positive correlation, but there are some discrepancies.

Given that the above correlation was computed for all the genes (Figure 4a), we next asked whether the correlation changed when we examined the essential/nonessential genes that we predicted using our machine learning approach. Interestingly, the correlation coefficient between the essentiality score and gene expression for the predicted essential genes in shRNA-treated plants averaged −0.304, while the correlation coefficient of CRISPR-treated plants averaged −0.435. These findings indicated that the essential genes identified by both shRNA and CRISPR had greater negative correlation coefficients between essentiality scores and expression values than did the correlation coefficients for all the genes (Figure 4b). Of particular interest, for all the nonessential genes, the correlation coefficients between essentiality and expression were approximately 0 for both the shRNA and CRISPR systems, indicating that the essentiality score of nonessential genes was not correlated with gene expression for either the shRNA or CRISPR system (Figure 4b). In summary, the essentiality scores of essential genes are negatively correlated with their gene expression, and this correlation is stronger for CRISPR than for shRNA. However, nonessential genes were not correlated with either shRNA or CRISPR.

### 3.4. shRNA Outperforms CRISPR in the Identification of Essential Genes with Low Expression Levels

**Figure 5:**
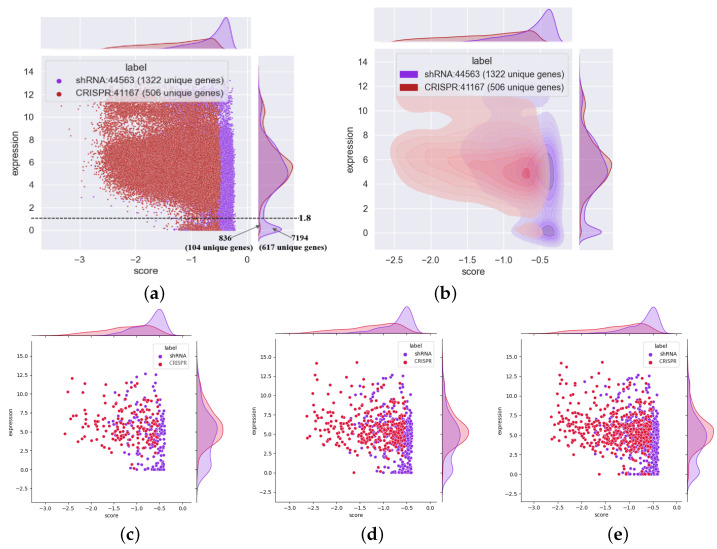

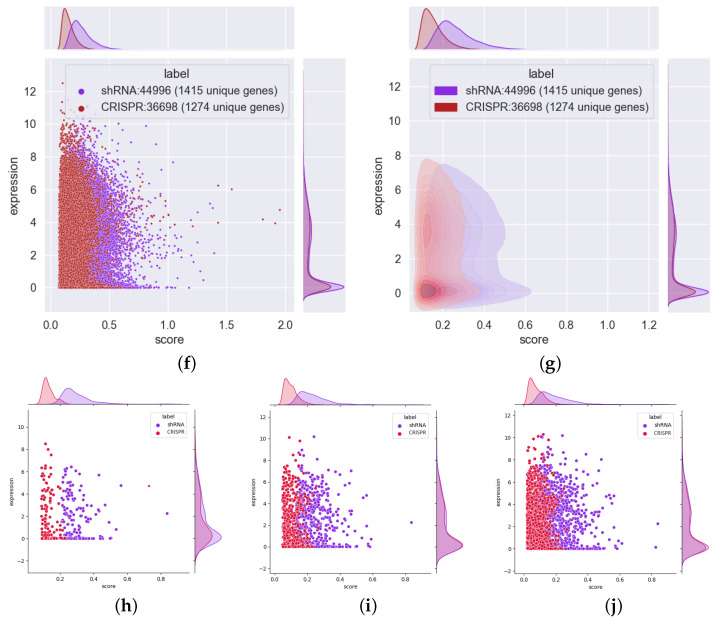
Scatterplot of the gene essentiality score and gene expression with marginal density plots and the kernel density estimate. (**a**,**b**) are based on all the essential genes with differential expression in 254 cell lines; (**c**–**e**) are based on the essential genes in the breast cancer cell line ACH-001819 with different *x*% parameters; (**f**,**g**) show all the nonessential genes with different gene expression; (**h**,**i**), and (**j**) are based on the nonessential genes from the breast cancer cell line ACH-001819 with different *x*% parameters. (**a**) All the essential genes; (**b**) kernel density estimate of all the essential genes; (**c**) essential genes (*x*% = 10%); (**d**) essential genes (*x*% = 20%); (**e**) essential genes (*x*% = 30%); (**f**) all the nonessential genes; (**g**) kernel density estimate of all nonessential genes; (**h**) nonessential (*x*% = 10%); (**i**) nonessential (*x*% = 20%); (**j**) nonessential (*x*% = 30%).

Having predicted common essential genes using an unsupervised learning model, we applied this information to correct false positives. In addition, to maintain the true positive rate, we took the intersection between genes from the top *x*% of the original essentiality score in each of 254 cell lines and the top *x*% of predicted common essential genes with a high estimated probability as the final essential genes of each cell line to conduct the next analysis. Then, we compared the gene expression distributions of all the essential genes identified by shRNA and CRISPR in 254 cell lines. The final number of essential genes is associated with parameter *x*%, and a small parameter indicates the high accuracy of the essential genes. To maintain the high accuracy of the essential genes in each cell line, we set *x* to 10. This resulted in 506 unique (41,167 with overlap) essential genes from CRISPR and 1322 unique (44,563 with overlap) essential genes from shRNA. A scatterplot of the essentiality scores and the expression of these essential genes with marginal density plots is shown in Figure 5a. According to the marginal density of gene expression, shRNA can identify many more genes with low expression than CRISPR. The density plot of shRNA has two peaks: one is in the low-expression area where CRISPR does not have this peak, and the other is in the much higher gene expression area where CRISPR also has a similar peak. In other words, shRNA performed much better than CRISPR in identifying genes associated with low expression. Specifically, shRNAs identified 617 unique genes (7194 with overlap). For essential genes (16%), the log2-transformed transcript count per million (TPM) expression was lower than 1.8, while for those whose expression was lower than 1.8, only 104 unique (836 with overlap) essential genes (2%) were identified. Therefore, we recommend using shRNA rather than CRISPR to identify essential genes when gene expression is lower than 1.8. To further visualize the differences in essential gene expression distributions, Figure 5b shows the kernel density estimates for all the essential genes determined via shRNA and CRISPR. It is apparent that shRNA has two density kernels, one in the low-expression area and the other in the high-expression area. However, CRISPR has only one density kernel in the high-expression area. Figure 5b further demonstrates the conclusion from Figure 5a. To determine whether this trend changed with increasing parameter *x*%, we used the breast cancer cell line ACH-001819 as an example with different parameters (10%, 20%, and 30%). Figure 5c–e show scatterplots with marginal density plots. With an increase in the parameter *x*%, the expression of the essential genes in this cell line increased, but the marginal density plots exhibited almost no changes. All of these studies revealed that shRNA showed much better performance in identifying genes expressed at low levels than CRISPR.

Similarly, we obtained the nonessential genes in each cell line for shRNA and CRISPR based on the predicted common nonessential genes and the original essentiality scores. With respect to shRNA, we obtained 1415 unique (44,996 with overlap) nonessential genes in 254 cell lines, whereas with respect to CRISPR, we obtained 1274 unique (36,698 with overlap) nonessential genes in 254 cell lines. Figure 5f,g show the scatterplot with marginal density plots and the kernel density estimates for all the nonessential genes. The results demonstrated that the nonessential genes identified via shRNA and CRISPR had almost the same trend in gene expression. The only difference is that most of the nonessential genes identified via CRISPR have lower essentiality scores than the nonessential genes identified via shRNA. This may be caused by differences in the intrinsic characteristics of the two screening techniques used. To further determine the influence of parameter *x*%, we set *x*% to different values (10%, 20%, and 30%), and the trend in gene expression remained the same for both the shRNA and CRISPR systems. Compared to those of the essential genes, most of the nonessential genes were enriched in the low gene expression area. In summary, shRNA performs better in identifying genes with low expression than CRISPR; however, for nonessential genes, both screening techniques maintain the same trend in differential gene expression. The essential and nonessential characteristics of each gene identified by CRISPR and shRNA are shown in Supplementary Table S3, which can provide a reference for the choice of these two screening techniques in different cell lines. In addition, the sample tissue type for each cell line is shown in Supplementary Table S4.

### 3.5. Most Essential Genes Are Highly Expressed, and CRISPR Has a Slightly Better Ability to Identify Highly Expressed Essential Genes

**Figure 6:**
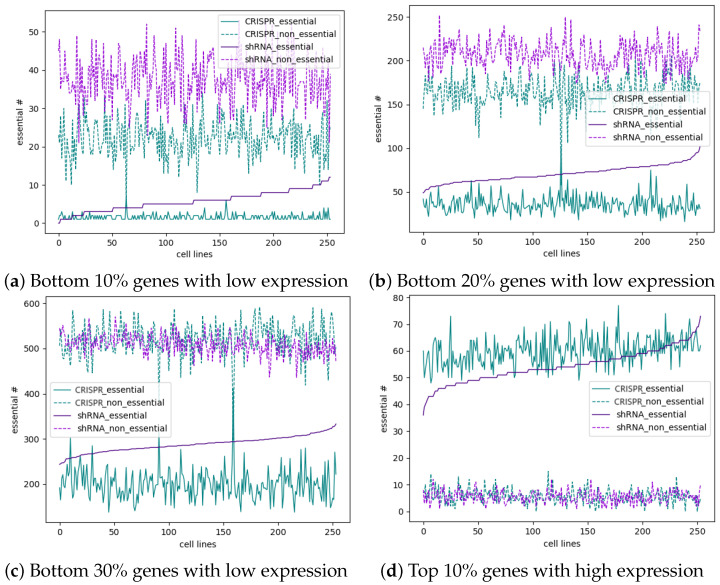

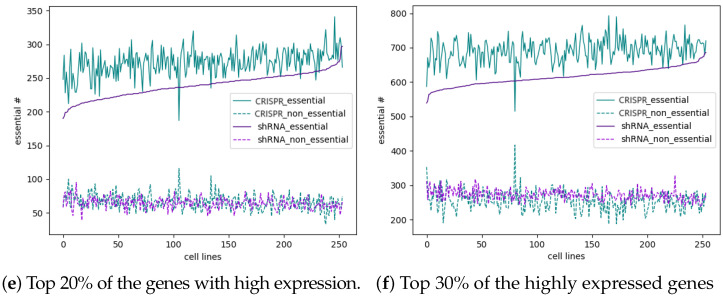
Comparison of the number of highly expressed essential (nonessential) genes and genes with low expression of essential (nonessential) genes identified by shRNA and CRISPR in each cell line. (**a**–**c**) show the number of essential and nonessential genes identified by shRNA and CRISPR in the top 10%, 20%, and 30%, respectively, of the genes with the lowest expression in each cell line. (**d**–**f**) show the numbers of essential genes and nonessential genes identified by shRNA and CRISPR in the top 10%, 20%, and 30%, respectively, of the genes most highly expressed in each cell line.

To further observe the ability of shRNA and CRISPR to identify highly expressed and lowly expressed essential genes, we focused on the high- and low-expression genes in each cell line. Figure 6 shows the number of essential and nonessential genes identified by shRNA and CRISPR among the genes expressed at low or high levels in each cell line. The purple color represents shRNA, and the yellow color represents CRISPR. The solid line represents essential genes, and the dotted line represents nonessential genes. As shown in Figure 6a, in the bottom 10% of genes with low expression, shRNA could be used to identify approximately 10 essential genes in each cell line, whereas CRISPR could be used to identify only approximately one essential gene. Additionally, shRNA can be used to identify approximately 40 nonessential genes, whereas CRISPR can only identify approximately 20 nonessential genes. These findings indicate that shRNA has much better performance in identifying both essential and nonessential genes than does CRISPR for genes with low expression. For the bottom 20% of genes with low expression shown in Figure 6b, both shRNA and CRISPR again identified more essential or nonessential genes than those in Figure 6a, but shRNA had better performance than CRISPR. When the percentage of genes with low expression increased to 30%, as shown in Figure 6c, the number of essential genes identified by shRNA and CRISPR increased to 300 and 200, respectively, but the number of nonessential genes identified by these two techniques was approximately 500. In contrast, for the top 10% of highly expressed genes in each cell line shown in Figure 6d, the number of identified nonessential genes was approximately five for both the CRISPR and shRNA, but the number of essential genes was slightly greater for the CRISPR system than for the shRNA system. For the top 20% and top 30% of highly expressed genes shown in Figure 6e,f, both screening techniques identified a similar number of nonessential genes, but CRISPR can identify a few more essential genes than can shRNA. Additionally, both screening techniques can identify more essential genes than nonessential genes among the highly expressed genes. In conclusion, shRNA has better performance than CRISPR in identifying both essential and nonessential genes that are expressed at low levels, whereas CRISPR has slightly better performance in identifying highly expressed essential genes. For identifying highly expressed nonessential genes, both techniques have similar performances. Among the genes expressed at low levels in each cell line, both screening techniques can identify more nonessential genes than essential genes, whereas among the highly expressed genes, both screening techniques can identify more essential genes than nonessential genes. When comparing the number of essential genes (or nonessential genes) among the lowly expressed genes and highly expressed genes, we find among the high-expression genes, there are more essential genes than among low-expression genes, but among the low-expression genes, there are more nonessential genes than among the high-expression genes. This finding is consistent with the previous conclusion that most of the essential genes are highly expressed [19,22]. We can also conclude that most of the nonessential genes have a greater probability of being expressed at low levels.

### 3.6. The Essential Genes Identified via shRNA and CRISPR Have Little Overlap, and Most of the Genes Expressed at Low Levels can Be Identified Only by shRNA

**Figure 7:**
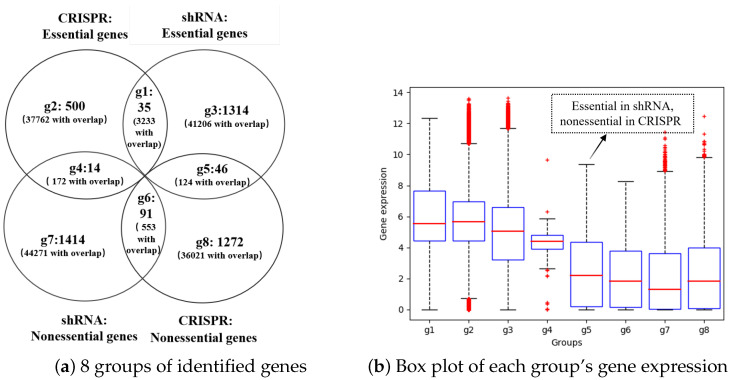
All the essential and nonessential genes identified by shRNA and CRISPR were compared. (**a**). All the identified genes were grouped into eight groups. Group 1 included genes that were both essential for CRISPR and shRNA; group 2 included genes that only showed essentiality for CRISPR; group 3 included genes that only showed essentiality for shRNA; group 4 included genes essential for CRISPR but nonessential for shRNA; group 5 included genes essential for shRNA but nonessential for CRISPR; group 6 included genes nonessential for both shRNA and CRISPR; group 7 included genes that only showed nonessential for shRNA; and group 8 included genes that only showed nonessentiality for CRISPR. (**b**). Box plot of the gene expression in each group. g1, g2, g3, g4, and g5 are essential groups, while g6, g7, and g8 are nonessential groups.

As previous studies have shown that shRNA and CRISPR use different biological processes with different GO term enrichments in essential complexes [10], we compared all the essential and nonessential genes identified by shRNA and CRISPR. To maintain the accuracy of the identified essential and nonessential genes, we set x% to 10%. Then, after gene knockdown with shRNA, 1322 unique (44,563 with overlap) essential genes and 1415 unique (44,996 with overlap) nonessential genes were obtained from 254 cell lines, and 506 unique (41,167 with overlap) essential genes and 1274 unique (36,698 with overlap) nonessential genes were also obtained from the knockout by CRISPR. Because each gene has a different expression value and essentiality in different cell lines, we determined the unique gene number, but we also counted duplicate genes with different gene expression levels and different essentiality scores in the different cell lines. We grouped all the essential genes and nonessential genes into eight groups according to overlap, as shown in Figure 7a. Group 1 included 35 unique (3233 with overlap) essential genes that were essential for both shRNA and CRISPR. Group 2 included 500 unique (37,762 with overlap) essential genes that were essential for CRISPR but irrelevant to shRNA. Group 3 included 1314 unique (41,206 with overlap) essential genes that were essential for shRNA but irrelevant to CRISPR. Group 4 included 14 unique (172 with overlap) genes that are essential for CRISPR but nonessential for shRNA. Group 5 included 46 unique (124 with overlap) genes that were essential for shRNA but nonessential for CRISPR. Group 6 included 91 unique (553 with overlap) genes that were nonessential for both shRNA and CRISPR. Group 7 included 1414 unique (44,271 with overlap) genes that were nonessential for shRNA but irrelevant to the CRISPR system. Group 8 included 1272 unique (36,021 with overlap) genes that are nonessential according to the CRISPR system but irrelevant to the shRNA.

We further compared the gene expression values in each group via box plots, which are shown in Figure 7b. The first five groups were essential genes, whereas the last three groups were nonessential genes. The first four essential gene groups had a median gene expression value of approximately 6, whereas the nonessential gene groups had a median expression value of approximately 2. It is apparent that essential genes usually have much higher gene expression levels than nonessential genes. The fifth group comprises the essential genes identified by shRNA but identified as nonessential genes by CRISPR. Even though they are essential genes, their gene expression levels are as low as those of nonessential genes. This further demonstrated that shRNAs can be used to identify genes whose expression is low compared with that of CRISPR. The essential genes identified only by CRISPR and put into group 2 had the highest median gene expression values, and the essential genes identified only by shRNA and group 3 had relatively lower median gene expression values. In summary, CRISPR and shRNA can be used to identify different essential and nonessential genes in each cell line, and the identified essential genes exhibit markedly greater expression than the identified nonessential genes. In addition, essential genes with low expression can be identified only by shRNA because they are usually misidentified as nonessential genes by CRISPR.

## 4. Conclusions and Discussion

shRNA and CRISPR are two effective screening platforms for evaluating a wide variety of gene functions and analyzing biological processes. These two screening technologies are also widely applied to screen essential genes in cancer, which helps with understanding the mechanism of disease and developing drugs. Previous studies have shown that both platforms can be used to effectively separate essential and nonessential genes with high precision, but the results of these two systems differ by only a small overlap. Even though the reasons for these differences are clear, including differences in off-target effects, heterogeneity of reagents, and intrinsic differences, it is unclear how the choice of these two systems affects the results. Many researchers have compared these two techniques and attempted to determine which has a better performance in essential gene detection. However, there are many limitations to the previous comparison. First, their conclusions are not consistent; some research has concluded that CRISPR has a better performance than shRNA, whereas others have concluded that the combination of CRISPR and shRNA has a better performance. Moreover, the studies that derived conclusions only based on one cell line with limited essential genes but different cell lines that have different essential genes and different gene expression and an insufficient number of essential genes cannot give a comprehensive conclusion. In addition, they ignore the gene expression level when they compare these two screening techniques, whereas previous studies have shown that essentiality should be highly associated with the gene expression level. Finally, those studies are based directly on the results of CRISPR and shRNA, both of which have a high false positive rate. Traditionally, the essentiality score is determined for all genes from the same cell line, and the genes in the lowest quartile are considered to be essential genes in that cell line. However, this method has two limitations: (1) the high false positive rate caused by the off-target effects of screening techniques means that the score cannot entirely represent the essentiality of the genes; and (2) the results are sensitive to the cutoff threshold. To address the first limitation, we propose a graph-based unsupervised learning model to identify the common essential genes that are expected to mitigate the false positive rate based on the conditional probability of each gene being essential. To address the second limitation, we combined the cutoff method with the graph-based machine learning model, which expects to maintain the true positive rate and alleviate the sensitivity with the cutoff values. Therefore, in this study, we first applied a graph-based unsupervised learning model to correct false-positive samples, and then we performed a synthetic comparison between CRISPR and shRNA screening platforms for the identification of essential genes at different gene expression levels in 254 matching cell lines.

The systematic comparison revealed that (1) shRNA has much better performance than CRISPR in identifying essential genes with low expression, and according to our analysis, we suggest using shRNA when the log2-transformed TPM expression value is lower than 1.8. (2) Most of the essential genes had a greater probability of being highly expressed, whereas nonessential genes had a greater probability of having lower expression. Additionally, CRISPR works slightly better than shRNA for identifying highly expressed essential genes. Most of the essential genes identified by shRNA and CRISPR with log2-transformed TPM had a gene expression value above 4, while CRISPR could identify more essential genes than could shRNA. Nonetheless, most of the nonessential genes had a much lower log2-transformed TPM expression value of approximately 1, and shRNA and CRISPR almost exhibited no difference in identifying nonessential genes. (3) Essential genes identified with CRISPR had a greater correlation coefficient between the essentiality score and gene expression than did those identified with shRNA, whereas the nonessential genes identified with these two screening techniques did not have a correlation between essentiality and gene expression. (4) The essential and nonessential genes identified by these two screening techniques have little overlap, and essential genes with low expression can be identified only by shRNA and are usually identified as nonessential genes by CRISPR. (5) Not a single gene is essential for all cancer cell lines.

In summary, this study led to the novel and important conclusion that shRNA has much better performance than CRISPR in identifying genes with low expression levels. In addition, this study gave a comprehensive guide to which platform should be selected to validate the essentiality of different genes in different cell lines. We suggested using shRNA to identify essential genes when the log2-transformed TPM expression value is lower than 1.8 and combining shRNA and CRISPR for more highly expressed essential genes. Compared with previous studies, this research had two advantages: we considered the high false positive rate of CRISPR and shRNA by using a graph-based unsupervised learning method to correct false positives, and our research was based on genes in all 254 cell lines, which avoided bias in an individual cell line; moreover, our conclusion was more confident than conclusions based on only one cell line. Generally, more cell line data should help improve the generalization, robustness, and confidence of the graph-based machine learning model. Therefore, in the future, we will keep collecting different cell lines’ data and train this model again with more datasets. In addition, we will further validate our conclusions via biological experiments, and our experiments will focus on genes whose expression is low and can be identified only by shRNA.

## Data Availability

The code and dataset of the model are available at https://github.com/XYDBCS/shRNACRISPR, accessed on 1 February 2024.

## Supplementary Materials

The following supporting information can be downloaded at: https://www.mdpi.com/article/10.3390/cells13191653/s1.
